# Sanitary Expenditure and Local Indebtedness

**Published:** 1903-01-17

**Authors:** 


### Sanitary Expenditure and Local Indebtedness.

In the recently-issued report of the Local Government Board a point is worked out with considerable elaboration which is of the greatest importance in all that concerns sanitary administration, namely, the steady and one might almost say the alarming growth of local indebtedness, for it is by aid of this borrowed money that most of the sanitary improvements of which we boast have been paid for, and if this well should run dry woe betide all sanitary progress. Sanitation costs money, and unfortunately the material improvements upon which a good deal of this money is spent are of a more or less ephemeral nature; in some cases they tend to wear out, in others they go out of fashion. Thus it happens that at the end of a comparatively small number of years we cannot be said to have a very solid asset in return for much of the money that has been expended, and there is a danger lest when the country becomes thoroughly awake to the fetters of debt which are being forged by us for succeeding generations, it may be to sanitary matters that the pruning knife will be applied and it may be by limiting the speed of sanitary advance that economists will endeavour to make both ends meet.

We are strongly of opinion that in improved conditions of life we get our full pennyworth for judicious expenditure upon sanitary matters; nevertheless, we cannot deny that the grumbler has a good deal to say for himself, and that the enormous growth not only of the rates levied for local purposes but of the outstanding debt which has been incurred by local authorities is a very serious matter. Indeed, but for the fact that most of this borrowing is for limited terms, and that automatic repayment is arranged for by appropriate sinking funds, the prospect for our successors would be a very gloomy one—and for a large proportion of this expenditure sanitation has to bear the blame.

Time was, not so very long ago, when a national Budget exceeding £100,000,000 was regarded as unexampled extravagance. Yet now, according to the report of the Local Government Board, in addition to all our national expenditure, no less than £100,862,280 is required in the course of a year for local expenditure alone. The report shows that between the years 1875 and 1900 the aggregate amount of public rates raised locally in England and Wales, increased by 112·2 per cent. But meanwhile the system of receiving grants from the central exchequer had greatly extended, so that during the same period the grants towards local expenditure raised by Imperial taxation had increased by no less than 628·5 per cent. Notwithstanding all this, however, so far have the local authorities been from managing to make both ends meet, that they have been constantly running more and more into debt. It is a startling thing to find that all our efforts, exerted during so many years, to ensure a gradual paying off of the National Debt are being far more than neutralised by the repeated borrowings of our local authorities. According to the figures given in the above-mentioned report, no less than 23 millions out of the 100 millions spent by the local authorities was raised by loan in the course of the year. Whether we look at the relief of the poor, at highways, at waterworks, gasworks, or sewerage, or at half a dozen other main spending departments, the same tale is told that far more—in some cases many times more—is being spent than is being raised by rates, notwithstanding the great increase in these rates that has taken place; we are outrunning our income in every direction.

If now we pass from current annual expenditure, which is more or less under annual control, and turn to the outstanding local loans, we find what a tangle of debt the country has got itself into. Between the year 1875 and 1900 the National Debt decreased by £139,966,974. Meanwhile, however, the outstanding loans of the local authorities had increased by £201,044,124. Thus, taking the debt as a whole, we are worse off than ever. Our local indebtedness has increased by 216 per cent., while our rateable value has only increased by 51 per cent. and our population by only 34 per cent. Now if we ask for what purposes all this borrowing has taken place, we find that it is distributed over nearly every item of local expenditure, but that nearly half of it is for items which may roughly be classed, in part or in whole, as coming under the head of sanitation—waterworks, highways and street improvements, sewerage and sewage disposal, parks and open spaces, hospitals, cemeteries, baths and wash houses—and one cannot but fear lest when the economist arises and the squeeze comes it will be sanitary matters which will go to the wall. Hence the supreme importance of giving him no excuse, and of exercising such care and matured judgment in regard to all sanitary expenditure that no one shall be able to accuse our health officials of recklessness or extravagance, lest perchance sanitation should come to stink in the nostrils of the people, and money for sanitary purposes should be refused.

